# A New Silver Alloy

**Published:** 1869-11

**Authors:** 


A New Silver Alloy, under the name of *alliage tiers-argent*, containing two-thirds of nickel and one-third silver, has been introduced by Alf. Jaloureau and sold by Musset (116 rue Rivoli), Paris, which challenges in many respects alloys of greater cost. It is used for plate especially; and as it possesses far greater hardness than richer alloys, and yields in like proportion to the chisel, will no doubt, meet with success. The price per kilo. is 90 francs, and, on returning, 75 francs are allowed.

